# Exploring the Link Between Extreme Weather Events and Prevalence of Mental Health Conditions

**DOI:** 10.1192/bjo.2025.10110

**Published:** 2025-06-20

**Authors:** Peter Carter, Amna Illahi, Fayed Iqbal, Itbaan Husaain, Callaghan Freya

**Affiliations:** Anglia Ruskin University, Chelmsford, United Kingdom

## Abstract

**Aims:** Extreme weather events refer to weather events that are dramatically different from typical patterns. These can be catastrophic, unexpected and pose a risk to the population. This review aims to examine whether sufficient evidence exists to demonstrate a link between extreme weather events and an increase in mental health conditions, specifically PTSD, anxiety, and depression.

**Methods:** We conducted a literature search across multiple electronic databases, including PubMed, Web of Science, Scopus, and PsycINFO, for articles published between January 2000 and January 2025. Keywords include Extreme weather; Mental health; Depression; Anxiety; Post traumatic stress disorder. From this we used four articles reporting quantitative data on the prevalence of mental health conditions in those exposed to extreme weather events. The selection of these four articles is justified based on the relevance to our research question. They provide figures which allow us to compare mental health prevalence before and after extreme weathers took place. Furthermore, they offer a vast array of data, from various populations to weather patterns, enabling us to conduct more thorough research.

**Results:** All four studies reported a rise in PTSD, anxiety, depression following extreme weather events like floods, heatwaves, and wildfires. A meta-analysis of surveys that targeted people who had been affected by floods in the previous 12 months found that the prevalence rates of anxiety (25.2%), depression (26.3%), and PTSD (30.4%) were generally higher in this group than in the overall population. The prevalence of anxiety (5.7%) and PTSD (7.8%) in the overall population was considerably lower than this. In contrast, depression did not experience such a large spike (20.6%). However, a cross-sectional analysis aiming to assess the relationship between flooding and psychological morbidity concluded that those experiencing cumulative and repeated exposure to extreme weather events such as flooding had a significant increased risk of developing depression but did not impact the levels of anxiety or PTSD. Two studies highlighted vulnerable groups including older adults and those with pre-existing mental health conditions are more susceptible to experience deteriorating symptoms.

**Conclusion:** Overall, there is sufficient evidence to highlight the strong association between extreme weather leading to an increase in prevalence of mental health conditions. These findings emphasize the urgent need for mental health support and early intervention strategies for the communities affected.

